# SGLT2 Inhibitor and GLP-1 Receptor Agonist Prescriptions in Newly Diagnosed Type 2 Diabetes Patients With Cardiorenal Risks: A Cross-Sectional Study

**DOI:** 10.1155/jdr/6656982

**Published:** 2025-11-03

**Authors:** Frank Müller, Michael J. Bouthillier, Omayma Alshaarawy, Hend Azhary, Harland T. Holman

**Affiliations:** ^1^Department of Family Medicine, College of Human Medicine, Michigan State University, Grand Rapids, Michigan, USA; ^2^Corewell Health Family Medicine Residency Center, Corewell Health, Grand Rapids, Michigan, USA; ^3^Department of General Practice, University Medical Center Göttingen, Göttingen, Germany; ^4^Department of Family Medicine, College of Human Medicine, Michigan State University, East Lansing, Michigan, USA

## Abstract

**Aims:**

The aim of this study is to evaluate the real-world prescribing patterns of SGLT2 inhibitors and GLP-1 receptor agonists (GLP-1RA) in patients with newly diagnosed Type 2 diabetes (T2DM), particularly among those with high cardiovascular risks or chronic kidney disease, and to identify demographic, clinical, and system-level factors associated with receiving these medications.

**Materials and Methods:**

This cross-sectional study analyzed electronic medical records (EMRs) of patients with newly diagnosed T2DM from 60 primary care clinics in West Michigan between April 2021 and January 2023. We assessed the documented prescription rates of SGLT2 inhibitors and GLP-1RAs within 3 months of diagnosis based on EMRs, particularly in high-risk subgroups.

**Results:**

Overall, 19.9% of *n* = 5783 patients with newly diagnosed T2DM had either an SGLT2 inhibitor or GLP-1RA prescribed. Prescription rates for these agents were 20.0% for patients with chronic ischemic heart disease and 19.3% for those with impaired kidney function. In adjusted analyses, higher BMI (aOR 2.92 for BMI > 40 kg/m^2^, 95% CI 1.58–5.42, ref BMI < 24 kg/m^2^), hyperlipidemia (aOR 1.89, 95% CI 1.28–2.79), chronic ischemic heart disease (aOR 1.55, 95% CI 1.11–2.18), and higher HbA1c (aOR 1.32 per 1% increase, 95% CI 1.22–1.42) were associated with higher odds of receiving prescription of these medications.

**Conclusions:**

Despite guideline recommendations, SGLT2 inhibitors and GLP-1RAs are prescribed to only a minority of patients with newly diagnosed T2DM, even among those with clear indications. Efforts to improve guideline-adherent care in primary care settings are needed.

## 1. Introduction

In the last decade, the management of Type 2 diabetes (T2DM) has evolved significantly with the introduction of novel antidiabetic medications, notably sodium–glucose cotransporter 2 (SGLT2) inhibitors and glucagon-like peptide 1 receptor agonists (GLP-1RAs). These agents have demonstrated benefits beyond glycemic control, including risk reduction for cardiovascular events and protection of kidney function [1, 2].

The American Diabetes Association (ADA) and the European Association for the Study of Diabetes (EASD) have updated their guidelines to reflect the growing evidence supporting the use of these newer medications. Starting in 2018, these organizations recommended the use of SGLT2 inhibitors or GLP-1RAs for patients with established cardiovascular disease, heart failure, or chronic kidney disease [3, 4]. Similar recommendations have been issued by cardiology associations [5, 6]. The 2021 ADA Standards of Medical Care in Diabetes further refined these recommendations, emphasizing the importance of SGLT2 inhibitors and GLP-1RAs in specific high-risk patient populations [7].

Despite these guideline updates, the translation of evidence-based recommendations into clinical practice often lags to the disadvantage of those under high cardiorenal risk, a phenomenon that has been described as “clinical inertia” [8]. Previous studies have identified various barriers to the effective management of T2DM in primary care settings, including limited time and resources, coverage and out-of-pocket costs, lack of confidence in knowledge, and uncertainty about clinical responsibilities [9–11].

In the United States, insurance coverage has been described as a primary determinant of access to SGLT2 inhibitors and GLP-1RAs due to their high costs. Medicare, Medicaid, and private insurance each implement distinct formulary restrictions, prior authorization requirements, and cost-sharing structures that can create significant access barriers, with pronounced disparities affecting racial and ethnic minorities [12, 13]. Socioeconomic factors have been shown to be associated with lower uptake, even among patients with clear guideline-based indications [14].

Understanding the current prescribing patterns of novel antidiabetics in primary care settings is crucial for identifying potential shortcomings in diabetes care and may provide explanations for known inequalities in diabetes outcomes [15, 16].

Given the evolving landscape of diabetes management and the potential gap between guideline recommendations and clinical practice, this study has two primary aims: (1) to evaluate the real-world prescribing patterns of SGLT2 inhibitors and GLP-1RAs in newly diagnosed T2DM patients with established cardiorenal risk factors and (2) to identify patient characteristics, clinical factors, and system-level factors associated with receiving prescriptions for these medications. We focused on newly diagnosed patients to evaluate guideline implementation at the critical point of initial treatment decisions [17], minimizing the influence of confounders like clinical inertia or established treatment patterns that often affect the management of longstanding diabetes. For the first aim, we determined the proportion of patients receiving prescriptions for these medications within 3 months of diagnosis. For the second aim, we examined how patient demographics, clinical characteristics, and comorbidities predicted the likelihood of receiving these medications.

## 2. Materials and Methods

This is a cross-sectional study using EMR data from *n* = 60 primary care clinics including family medicine and internal medicine clinics in West Michigan. The study protocol was reviewed through the Corewell Health Institutional Review Board and was deemed non-human subject research (Decision# 2022-337). Presentation of study results adheres to STROBE guidelines for cross-sectional studies [18] (see supporting information for the STROBE checklist).

### 2.1. Setting

This study was conducted within the Corewell Health West system, a large nonprofit managed healthcare organization in West Michigan. The system comprises 22 hospitals and over 300 outpatient facilities. While most clinics are located in the Grand Rapids–Kentwood–Muskegon–Combined Statistical Area serving urban and suburban communities, a significant proportion is situated in more rural areas of Northern Michigan.

Data were collected from 60 primary care clinics, including both family medicine and internal medicine practices.

### 2.2. Sample

This study included all unique adult patients (aged ≥ 18 years) from 60 primary care clinics who had an encounter between April 1, 2021, and January 31, 2023, in which either (a) T2DM was newly diagnosed (ICD-10: E11) or (b) a first-ever HbA1c value exceeding 6.5% (48 mmol/mol) was recorded. Eligibility criteria included being an established patient with a primary care provider in the system, defined as having at least one prior visit with the same office. Patients were identified through electronic health record queries based on these inclusion criteria. Exclusion criteria were as follows: receiving a Type 1 diabetes diagnosis (ICD-10: E10) or gestational diabetes (ICD-10: O24.4) at the encounter or within 3 months after the index encounter, age < 18 years, ongoing prescribed medication of systemic glucocorticoids (ATC: H02A, H02B) exceeding 2.5 mg prednisone equivalent, diagnosis of Cushing's syndrome (ICD-10: E24), or being newly established in the clinic. Patients with prediabetes (ICD-10: R73.03) were included if they met other criteria. Eligibility criteria were applied during initial data extraction in accordance with data minimization principles.

### 2.3. Measures

We collected data on several potential confounders and effect modifiers based on their known associations with diabetes management and outcomes. Sociodemographic variables included age (in years, also categorized into groups: < 35, 35–49, 50–64, 65–79, and ≥ 80), sex, race and ethnicity, and type of health insurance (Medicare, Medicaid, private, or none).

Race and ethnicity were reported using a two-step approach to create mutually exclusive categories: First, ethnicity was determined based on whether individuals identified with Hispanic cultural or ethnic identity. All individuals identifying as Hispanics were categorized in this group regardless of their racial identification. Those not identifying as Hispanic were then categorized based on their self-reported race as non-Hispanic American Indian or Alaska Native, non-Hispanic Asian, non-Hispanic Black or African American, non-Hispanic White, or non-Hispanic other (including Native Hawaiian or other Pacific Islander). All racial categorizations were based on self-report, with individuals identifying with multiple races included in the non-Hispanic other category. This mutually exclusive categorization approach was employed to prevent multicollinearity in regression models that would arise from including race and ethnicity as separate covariates. While separate reporting is often recommended [19], the combined approach was necessary to avoid effect size underestimation, as many Hispanic individuals identify racially as “other” or “Black or African American”, causing effect partitioning across correlated predictors.

Clinical covariates included comorbidities such as hypertension (ICD-10: I10), hyperlipidemia (ICD-10: E78), chronic ischemic heart disease (ICD-10: I25), chronic (auto-)inflammatory conditions (ICD-10: D89.9, M30-M36, K50-K52, K75.4), malignancies (ICD-10: C00-D48), and depression (ICD-10: F32). These were identified using ICD-10 codes from the electronic medical records and were coded if patients received one of these diagnoses prior to inclusion.

We also collected data on body mass index (BMI, kilograms per square meter) measured in the clinics prior to encounters. Furthermore, we obtained serum creatinine and calculated the estimated glomerular filtration rate (eGFR, mL/min/1.73 m^2^) using the four-variable Modification of Diet in Renal Disease (MDRD) approach [20] (considering the patient's sex, ethnicity, and serum creatinine levels), as well as HbA1c (percent and millimoles per mole). These measures were extracted at inclusion and for the subsequent 3-month period. When multiple values were available, we calculated the mean to account for potential variability and delayed diagnostics. Decreased kidney function was determined exclusively through laboratory-measured serum creatinine values, not diagnostic codes. Patients were classified as having decreased kidney function if their calculated eGFR was < 60 mL/min/1.73 m^2^ based on the most recent creatinine measurement within the study period. Patients without available creatinine measurements were excluded from kidney function analyses.

HbA1c analyses were performed from whole blood EDTA by either two within-system laboratories using high-performance liquid chromatography (BioRad-D-100) or point-of-care tests. Serum creatinine was analyzed using enzymatic tests of blood collected in lithium heparin gel or serum separator SST gel container. All analyses were performed in one of 10 CLIA-certified in-system laboratories, with 24-h availability and typical 1-day turnaround time, ensuring adherence to the US federal quality standards and standardized testing.

Other studies have suggested renal protective effects of SGLT2 inhibitors in patients with highly impaired kidney function (with an eGFR down to 10 mL/min) [21]. Current guidelines, however, suggest using SGLT2 inhibitors in patients with eGFR ≥ 20 mL/min and GLP-1RAs in patients with eGFR ≥ 25 mL/min [22]. Thus, we have excluded individuals with an initial eGFR < 20 mL/min.

Similarly, antidiabetic drug prescriptions were documented at diagnosis and within the subsequent 3 months. This extended follow-up period accommodated various clinical scenarios: the gradual introduction of multiple medications, referrals to specialists for confirmatory diagnosis (e.g., to rule out Type 1 diabetes), and cases where patients initially hesitated to accept pharmacological treatment. Antidiabetic drug prescriptions were recorded regardless of the provider type or specialist who prescribed it.

Antidiabetic drugs were categorized as follows: DPP4 inhibitors (alogliptin, linagliptin, saxagliptin, and sitagliptin), SGLT2 inhibitors (canagliflozin, dapagliflozin, empagliflozin, and ertugliflozin), GLP-1RAs (exenatide, dulaglutide, liraglutide, semaglutide, and tirzepatide), insulin (various types including aspart, degludec, detemir, glargine, glulisine, lispro, NPH, and regular), metformin, glitazones (pioglitazone), and sulfonylureas (glimepiride, glipizide, and glyburide).

If patients were prescribed a combination of substances in one drug, they were accounted for in the categories of the respective substances (e.g., a combined formula of metformin and pioglitazone was counted for each category).

Missing values were described at each stage, and subsamples with more complete data were compared with the overall sample.

### 2.4. Outcomes

The primary outcome was defined as the prescription rates of either a GLP-1RA or a SGLT2 inhibitor within 3 months of initial diagnosis in newly diagnosed T2DM patients or hyperglycemic patients (HbA1c > 6.5%). The prescription rate was calculated as the number of patients with EMR documented prescription of these antidiabetics, divided by the total number of patients in the respective analysis group. We have assessed this outcome in (a) the overall study population, and among patients with certain risk factors, including (b) patients with decreased kidney function (eGFR < 60 mL/min/1.73 m^2^), (c) patients with diagnosed chronic ischemic heart condition (ICD-10: I25), and (d) patients with a BMI ≥ 32 kg/m^2^.

The prescription rate was defined as the proportion of all patients in each analysis group who had EMR-documented prescriptions for either an SGLT2 inhibitor or GLP-1RA within 3 months of their T2DM diagnosis. Patients receiving multiple prescriptions within this period were counted once.

The risk subgroups were derived from the Standards of Care in Diabetes from the ADA from 2021 [23] as well as the 2020 Expert Consensus on Novel Therapies for Cardiovascular Risk Reduction of the American College of Cardiology [6]. Throughout the study period, these guidelines maintained consistent key recommendations despite minor updates. They strongly advise the use of SGLT2 inhibitors or GLP-1RAs for people with T2DM with either chronic kidney disease (eGFR < 60 mL/min) and/or established atherosclerotic cardiovascular disease or multiple cardiovascular risk factors. In a further subgroup, we assessed prescription rates in patients with a BMI ≥ 32 kg/m^2^. This aligns with recommendations to consider diabetes-related risks and weight management goals, as favorable weight outcomes significantly improve glycemic control and cardiovascular risk. While current guidelines suggest considering the weight-reducing potential of antidiabetics for patients with obesity, they do not specify a clear BMI threshold for this recommendation. Our BMI threshold has been set pragmatically but was informed by study findings, for example, a relative risk of 1.5 for sudden cardiac death in people with a BMI of 32 kg/m^2^ [24].

### 2.5. Sample Size

For this observational study, a formal power analysis was not conducted prior to data collection. With including several thousand T2DM patients and similar substantial numbers in key subgroups, we anticipated having adequate statistical power to identify significant associations and conduct robust subgroup analyses, even when considering potential confounders and the need for adjusted analyses.

### 2.6. Statistical Analyses

We used descriptive statistics to characterize our sample, reporting absolute and relative frequencies for categorical variables, and means with standard deviations (SDs) for continuous variables. Bivariate analyses were conducted to compare characteristics between groups using chi-square tests for categorical variables and Mann–Whitney *U* tests for continuous variables.

To assess factors associated with the prescription of GLP-1RAs or SGLT2 inhibitors and understand potential drivers of prescribing variations, we performed logistic regression analyses. We first conducted unadjusted analyses, followed by multivariable models adjusting for potential confounders. These included demographic factors known to influence medication access (age, sex, race and ethnicity, and insurance type), clinical parameters that guide prescribing decisions (BMI, eGFR, and HbA1c), and comorbidities that might affect treatment choice or reflect overall disease burden [12, 25–27]. To adjust for provider and regional setting, we further adjusted for the specific clinic site of the primary care provider. Patients with missing values for key analysis variables were excluded using complete case analysis. Results are presented as either crude odds ratios (ORs) or adjusted odds ratios (aORs) with 95% confidence intervals (CI).

We conducted sensitivity analyses stratified by key subgroups, including patients with chronic ischemic heart disease, impaired kidney function (eGFR < 60 mL/min/1.73 m^2^), or severe obesity (BMI > 32 kg/m^2^). Given the clinical indications for SGLT2 inhibitors for heart failure, we conducted a sensitivity analysis examining factors associated with SGLT2 inhibitor prescribing specifically. All analyses were performed using SPSS 29 (IBM Corp., Armonk, NY), with a two-sided *p* value < 0.05 considered statistically significant.

## 3. Results

### 3.1. Sample Description

**Figure 1:**
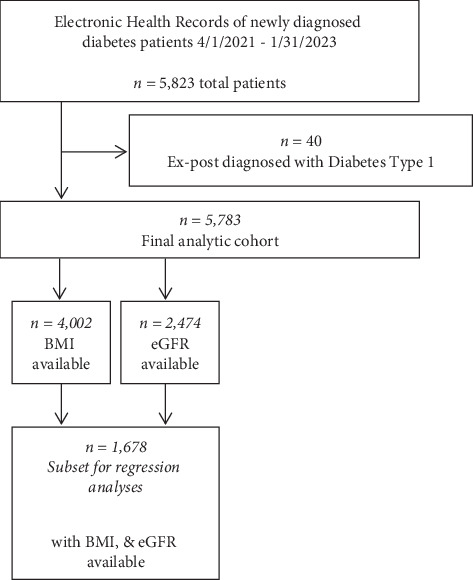
Flowchart of patient inclusion.

After applying exclusion criteria, *n* = 5783 patients were included in analyses. Data on heart conditions and HbA1c were available for all patients. BMI (*n* = 4002) and eGFR (*n* = 2474) were available for fewer of the patients; a subsample with both BMI and eGFR included 1678 patients (Figure 1). Each of the 60 clinics contributed on average data from 96.4 (SD 91.7) diabetes patients to the analyzed sample.

Patients in the sample were on average 62.0 years old (SD 14.5) and more often of male sex (56.3%) with a large proportion of non-Hispanic Whites (82.6%). Almost half of our sample were insured through Medicare (49.3%). The majority of enrolled patients had hyperlipidemia (80.3%) and hypertension (78.8%). Of the overall sample, 18.0% (*n* = 1036) had a diagnosed chronic ischemic heart condition, 20.7% (*n* = 511) had impaired kidney function (eGFR < 60, missing creatinine *n* = 3312), and *n* = 2286 or 57.1% of the subjects had a BMI of 32 or higher (missing BMI *n* = 1781). More characteristics are depicted in Table 1.

The study analyzed subgroups based on BMI and eGFR data availability. Patients with available BMI data were older (+0.9 years), more often non-Hispanic Whites (84.7% vs. 82.6% in the overall sample), less privately insured (38.7% vs. 40.4%), and had more often hyperlipidemia (81.6% vs. 80.3%) compared to the overall sample. Those with eGFR data were older (+0.5 years) and had more chronic ischemic heart conditions (20.7% vs. 18.0%) compared to the overall sample. A group of 1678 patients with both BMI and eGFR data, used for regression analyses, was older (+1.4 years), had higher Medicare coverage (52.9% vs. 49.3%), more chronic ischemic heart disease (21.6% vs. 18.0%), and cancer diagnoses (29.8% vs. 26.9%) compared to the overall sample. All differences were statistically significant (*p* < 0.05) and are shown in Table S1.

### 3.2. Diabetic Characteristics and Treatment

**Figure 2:**
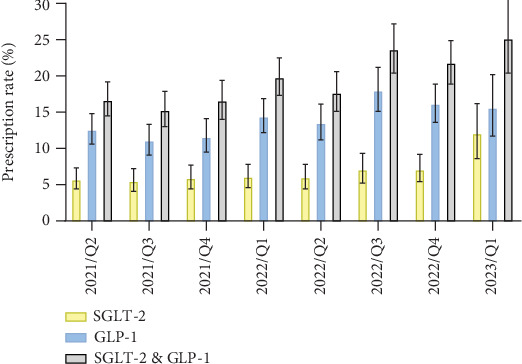
Prescriptions rates for GLP-1RAs and SGLT2 inhibitors over study quarters.

Among all included patients, 19.9% (*n* = 1092) were prescribed antidiabetic medication that included either a GLP-1RA or SGLT2 inhibitor, with increasing prescription rates during the observation period (2021: 16.2%, 2022: 20.6%, 2023: 25.2%; see also Figure 2).

Patients with chronic ischemic heart condition or impaired kidney function, who have established indications for these medications, received such prescriptions at rates of 20.0% and 19.3%, respectively, within 3 months of diagnosis or detection of increased HbA1c. These rates were not statistically significantly different from patients without these conditions (18.7% in those without heart condition, *p* = 0.333; 20.5% in those with normal kidney function, *p* = 0.547).

Prescription rates increased from 2021 to 2023 in both groups (heart condition: 17.6%–30.9%; impaired kidney function: 17.8%–28.6%) but remained similar to rates in patients without these conditions throughout each year (2021–2023: all *p* > 0.2).

Patients with severe obesity (BMI ≥ 32 kg/m^2^) were slightly more likely to receive prescriptions of these antidiabetics (20.3% vs. 15.3% in nonobese patients, *p* < 0.001). Prescription rates in this group increased from 18.3% in 2021 to 24.2% in 2023 and were significantly higher than in nonobese patients during 2021 (18.3% vs. 14.5%, *p* = 0.033) and 2022 (21.7% vs. 15.2%, *p* < 0.001), but not in 2023 (24.2% vs. 23.1%, *p* = 0.852). Additional diabetic characteristics are presented in Table 2.

In regression analyses of a sample with complete BMI and eGFR data (*n* = 1678), several factors showed statistically significant associations with receiving a prescription for GLP-1RAs or SGLT2 inhibitors. Age was significant in bivariate analysis (crude OR 0.98, 95% CI 0.97–0.99) but lost significance after adjustment (aOR 0.99, 95% CI 0.97–1.00). Similarly, the crude association with Medicare insurance (crude OR 0.62, 95% CI 0.48–0.80) weakened in the adjusted model (aOR 0.69, 95% CI 0.47–1.01).

Hyperlipidemia and chronic ischemic heart disease became significant only after adjustment (aOR 1.89, 95% CI 1.28–2.79 and aOR 1.55, 95% CI 1.11–2.18, respectively). The association between BMI and medication prescription strengthened after adjustment, particularly for BMI > 40 (crude OR 2.54, *p* = 0.001; aOR 2.92, *p* = 0.001) and BMI 35–39.9 (crude OR 1.81, *p* = 0.037; aOR 2.13, *p* = 0.016). HbA1c remained a consistent predictor in both crude (OR 1.29, 95% CI 1.21–1.38) and adjusted (aOR 1.32, 95% CI 1.22–1.42) analyses. Sex, race and ethnicity, and eGFR showed no significant associations in the adjusted model; however, some racial/ethnic categories showed a statistically significant impact on prescription of newer antidiabetics in bivariate analysis. All ORs are displayed in Table 3.

Additional regression analyses were performed separately for key subgroups: patients with chronic ischemic heart disease, impaired kidney function, and severe obesity (Table S2). In further sensitivity analyses examining SGLT2 inhibitors separately (rather than combined with GLP-1RAs, aORs omitted) among patients with chronic ischemic heart disease, most associations differed from those seen in the combined medication analysis. While the subgroup analysis in Table S2 showed significant associations with BMI 25–29.9 (aOR 5.66, 95% CI 1.03–31.23) and HbA1c (aOR 1.46, 95% CI 1.14–1.87), when examining SGLT2 inhibitor prescriptions alone, no statistically significant predictors were identified when examining SGLT2 inhibitor prescriptions alone.

In a sensitivity analysis examining SGLT2 inhibitor prescribing specifically, 371 (6.4%) of 5783 patients were prescribed SGLT2 inhibitors. SGLT2 prescribing rates were higher among patients with cardiovascular comorbidities: 9.2% of patients with chronic ischemic heart disease received SGLT2 inhibitors compared to 5.8% of those without. In multivariable analysis (Table S3) adjusting for various clinical and sociodemographic factors, this finding was confirmed: patients having chronic ischemic heart disease (aOR 2.46, 95% CI 1.52–3.96) had significantly higher odds of receiving SGLT2 inhibitors than those without this condition.

## 4. Discussion

Our study showed that 19.9% of newly diagnosed T2DM received a prescription for GLP-1RA or SGLT2 inhibitor within 3 months of diagnosis/elevated HbA1c. More concerning, among patients with chronic ischemic heart disease or impaired kidney function, who have clear indications for these medications [7], prescription rates were only marginally higher at 20.0% and 19.3%, respectively. Patients with severe obesity (BMI ≥ 32 kg/m^2^) were slightly more likely to be prescribed these newer antidiabetics (20.3%). Higher SGLT2 inhibitor prescribing rates in patients with ischemic heart disease align with current guidelines, though absolute prescription rates in this population remain low with < 10%.

Our results corroborate findings of low utilization rates for newer antidiabetics in high cardiovascular risk diabetes patients [28, 29]. They are also consistent with several studies that have reported lower odds of receiving prescriptions for these medications among certain racial and ethnic groups [27].

Our findings may differ from these studies, as we focused on people newly diagnosed with T2DM, offering insights into initial treatment decisions. We chose to focus on newly diagnosed patients, who likely have fewer preconceptions about medications, to minimize confounding factors. This contrasts with individuals who have ongoing and established treatment regimens, whose previous experiences and preferences may make them hesitant to switch to another medication. Still, the discussion of injectable medications in diabetes treatment plans, perhaps even as lifelong medication, can be perceived as challenging, especially to patients who just learned about their new chronic and perhaps even life-changing condition. While historically most GLP-1RAs required subcutaneous injection that can be a significant barrier to patient acceptance, oral semaglutide has been introduced in 2019 [30].

GLP-1RAs are not novel therapies in the diabetes treatment landscape; exenatide, the first in this class, entered the US market in 2005. Similarly, the first SGLT2 inhibitors received approval by the FDA in 2013. Despite their long-standing availability and evolving evidence base, our findings suggest that providers may still primarily view these medications through a narrow lens of glycemic control. Our regression analyses indicate that higher HbA1c values are strongly associated with the prescription of these newer antidiabetic substance classes, implying that they are often reserved for patients with poorly controlled or hard-to-manage diabetes and thus neglecting their proven efficacy for reducing cardiorenal risks [2]. Other research has highlighted that primary care clinicians face challenges in keeping up with rapidly evolving diabetes management guidelines amid time constraints and competing priorities in primary care, possibly contributing to the underprescription of newer antidiabetics to high-risk individuals [31].

The recent and ongoing shortage of GLP-1RAs that has partly been attributed to lifestyle prescriptions for weight control may have influenced the prescribing patterns observed in our study [32]. As a result, in the United Kingdom, clinicians in the National Health Service (NHS) were suggested to stop prescribing GLP-1RAs for weight loss indications and avoid starting new GLP-1RA treatments for patients with T2DM [33, 34]. Following the current treatment guidelines, SGLT2 inhibitors are suggested to be considered as an alternative [35].

However, it is important to note that our study counted prescriptions and not medications dispensed to patients. Pharmacy-level shortages may have prevented some patients from receiving their prescribed GLP-1RAs, potentially leading to an overestimation of actual usage. Future studies should consider investigating both prescription and dispensing data to provide a more comprehensive picture of GLP-1RA utilization in the context of ongoing supply challenges.

Even before our study period, changes in Medicaid formulary coverage have significantly improved access to SGLT2 inhibitors and GLP-1RAs in Michigan. As of January 1, 2021, several SGLT2 inhibitors, including canagliflozin, dapagliflozin, and empagliflozin, were added to the Medicaid formulary lists as preferred agents. This change likely reduced out-of-pocket costs for patients and simplified the prescription process for providers. Similarly, Medicaid has expanded coverage for GLP-1RAs. Semaglutide was added to the Medicaid formulary list in 2020, followed by liraglutide in 2022, both as preferred agents [36]. Similarly, Medicare formulary coverage for SGLT2 inhibitors and GLP-1RAs has undergone substantial improvements in the last decade. A comprehensive analysis of Medicare formulary restrictions from 2019 to 2023 demonstrated dramatic reductions in coverage barriers; for example, by 2023, only 2.0% of SGLT2 inhibitor coverage policies had restrictions compared to 23.8% in 2019 [37].

Limitations of our study include reliance on EMR data, which may not reflect actual medication uptake or adherence, and inability to account for out-of-network prescriptions. However, in-system primary care providers likely renew external prescriptions, mitigating this issue. A notable limitation of our study was the lack of detailed socioeconomic data. While insurance status served as a rough proxy, more comprehensive information could have offered valuable insights, especially considering the substantial copayments for GLP-1RAs. These costs might influence prescription patterns, contrasting with SGLT2 inhibitors, which typically have lower out-of-pocket expenses. A major strength is the large sample size from 60 primary care clinics. Our focus on newly diagnosed patients provides valuable insights into initial treatment decisions for newer antidiabetic medications.

## 5. Conclusions

Our study demonstrates that patients with cardiorenal risk factors and a clear indication for SGLT2 inhibitors or GLP-1RAs receive prescriptions for these medications at suboptimal rates, indicating inadequate implementation of evidence-based guidelines in primary care. Education of providers should highlight the benefits of cardiovascular risk reduction, similar to statins, which are today primarily regarded as medications to reduce cardiovascular events rather than as medications to reduce high cholesterol levels.

## Figures and Tables

**Table 1:** Patient characteristics.

	**All (** **n** = 5783**)**	**Chronic ischemic heart condition (** **n** = 1036**)**	**Impaired kidney function (** **n** = 511**)**	**Severe obesity (** **n** = 2286**)**
	**n** ** (%)**	**n** ** (%)**	**n** ** (%)**	**n** ** (%)**
Sociodemographics				
Sex				
Male	3253 (56.3)	711 (68.6)	227 (44.2)	1242 (54.3)
Female	2530 (43.7)	325 (31.4)	287 (55.8)	1044 (45.7)
Age (years)				
Mean (SD)	62 (14.5)	71 (10.6)	72.7 (12.1)	60.6 (13.5)
< 35	228 (3.9)	0 (0)	4 (0.8)	88 (3.8)
35–49	883 (15.3)	25 (2.4)	16 (3.1)	373 (16.3)
50–64	1995 (34.5)	248 (23.9)	94 (18.3)	864 (37.8)
65–79	2047 (35.4)	530 (51.2)	237 (46.1)	808 (35.3)
80+	630 (10.9)	233 (22.5)	163 (31.7)	153 (6.7)
Race and ethnicity				
Hispanic (any race)	407 (7.1)	36 (3.5)	24 (4.7)	138 (6.1)
Non-Hispanic American Indian or Alaska Native	27 (0.5)	7 (0.7)	2 (0.4)	15 (0.7)
Non-Hispanic Asian	145 (2.5)	16 (1.6)	13 (2.5)	16 (0.7)
Non-Hispanic Black or African American	357 (6.2)	47 (4.6)	34 (6.7)	121 (5.3)
Non-Hispanic White	4736 (82.6)	913 (88.8)	433 (84.7)	1947 (85.9)
Non-Hispanic other^a^	64 (1.1)	9 (0.9)	5 (1)	29 (1.3)
Type of insurance				
None	68 (1.2)	3 (0.3)	1 (0.2)	16 (0.7)
Medicare	2853 (49.3)	800 (77.2)	419 (81.5)	1069 (46.8)
Medicaid	526 (9.1)	50 (4.8)	25 (4.9)	239 (10.5)
Other private insurance	2336 (40.4)	183 (17.7)	69 (13.4)	962 (42.1)
Comorbidities				
Hypertension	4547 (78.8)	985 (95.1)	487 (94.7)	1869 (82)
Hyperlipidemia	4642 (80.3)	989 (95.5)	467 (90.9)	1838 (80.4)
Chronic inflammatory condition	284 (4.9)	58 (5.6)	29 (5.6)	92 (4)
Chronic ischemic heart disease	1036 (18)	1036 (100)	175 (34)	395 (17.3)
Malignancy/cancer diagnosis	1553 (26.9)	393 (37.9)	206 (40.1)	608 (26.7)
Depression	1418 (24.6)	255 (24.6)	124 (24.1)	645 (28.3)

*Note:* Comorbidity subgroups are not mutually exclusive. Patients may belong to multiple groups if they meet multiple criteria (e.g., having both chronic ischemic heart disease and impaired kidney function). Chronic ischemic heart condition: I25; impaired kidney function: eGFR < 60 mL/min/1.73 m^2^; severe obesity: BMI > 32 kg/m^2^.

^a^Native Hawaiian, other Pacific Islander, and individuals identifying with multiple races.

**Table 2:** Diabetes characteristics.

	**All (** **n** = 5783**)**	**Chronic ischemic heart condition (** **n** = 1036**)**	**Impaired kidney function (** **n** = 511**)**	**Severe obesity (** **n** = 2286**)**
	**n** ** (%)**	**n** ** (%)**	**n** ** (%)**	**n** ** (%)**
BMI (kg/m^2^)				
Mean (SD)	34.1 (7.4)	33 (6.6)	33.7 (7.4)	38.9 (6)
< 25	320 (8)	72 (9.8)	31 (9)	—
25–29.9	963 (24.1)	201 (27.3)	87 (25.1)	—
30–34.9	1085 (27.1)	184 (25)	96 (27.7)	655 (28.7)
35–39.9	861 (21.5)	171 (23.2)	65 (18.8)	861 (37.7)
> 40	768 (19.2)	108 (14.7)	67 (19.4)	768 (33.6)
eGFR (mL/min)				
Mean (SD)	84.3 (30.9)	71 (28.9)	44.4 (12.5)	84.5 (30.5)
≥ 60	1960 (79.4)	334 (65.6)	—	801 (81.1)
45–59	298 (12.1)	87 (17.1)	298 (58.7)	109 (11)
30–44	143 (5.8)	55 (10.8)	143 (28.1)	51 (5.2)
< 30	67 (2.7)	33 (6.5)	67 (13.2)	27 (2.7)
Prescribed diabetes medication				
Metformin	2513 (43.5)	348 (33.6)	128 (24.9)	1045 (45.7)
Glitazones	299 (5.2)	41 (4)	25 (4.9)	135 (5.9)
Sulfonylurea	526 (9.1)	111 (10.7)	59 (11.5)	223 (9.8)
DPP4	186 (3.2)	38 (3.7)	22 (4.3)	61 (2.7)
SGLT2i	371 (6.4)	95 (9.2)	33 (6.4)	144 (6.3)
GLP-1	798 (13.8)	130 (12.5)	71 (13.8)	358 (15.7)
Insulin	742 (12.8)	152 (14.7)	108 (21.0)	263 (11.5)
Number of prescribed antidiabetics				
None	2075 (35.9)	443 (42.8)	209 (40.7)	786 (34.4)
1	2403 (41.6)	359 (34.7)	198 (38.5)	960 (42.0)
2	956 (16.5)	163 (15.7)	78 (15.2)	383 (16.8)
3+	349 (6.0)	6.9 (71)	29 (5.6)	157 (6.9)

*Note:* Subgroups are not mutually exclusive. Patients may belong to multiple groups if they meet multiple criteria (e.g., having both chronic ischemic heart disease and impaired kidney function). Chronic ischemic heart condition: I25; impaired kidney function: eGFR < 60 mL/min/1.73 m^2^; severe obesity: BMI > 32 kg/m^2^.

Abbreviations: DPP4, dipeptidyl peptidase-4; GLP-1, glucagon-like peptide-1 receptor agonists; SGLT2i, sodium–glucose cotransporter-2 inhibitor.

**Table 3:** Patient factors associated with SGLT2 inhibitor or GLP-1RA prescriptions in newly diagnosed T2DM.

**Factor**	**All (** **n** = 1678**)**			
	**Crude OR (95% CI)**	**p**	**aOR (95% CI)**	**p**
Age (cont.)	0.98 (0.97–0.99)	**< 0.001**	0.99 (0.97–1.00)	0.092
Sex				
Male	1.00 (0.78–1.27)	0.996	0.86 (0.65–1.14)	0.298
Female	Ref		Ref	
Race and ethnicity				
Hispanic (any race)	1.47 (0.94–2.29)	0.088	1.06 (0.63–1.79)	0.833
Non-Hispanic American Indian or Alaska Native	6.24 (1.04–37.53)	**0.045**	5.12 (0.79–33.16)	0.087
Non-Hispanic Asian	0.94 (0.41–2.16)	0.883	1.15 (0.46–2.85)	0.766
Non-Hispanic Black or African American	0.88 (0.50–1.56)	0.659	0.65 (0.34–1.26)	0.205
Non-Hispanic other	0.38 (0.09–1.62)	0.190	0.27 (0.06–1.26)	0.095
Non-Hispanic White	Ref		Ref	
Type of insurance				
None	1.87 (0.54–6.47)	0.324	2.29 (0.54–9.66)	0.261
Medicare	0.62 (0.48–0.80)	**< 0.001**	0.69 (0.47–1.01)	0.053
Medicaid	1.10 (0.73–1.67)	0.653	0.91 (0.57–1.47)	0.704
Other private insurance	Ref		Ref	
Hypertension	0.95 (0.70–1.28)	0.722	1.01 (0.71–1.46)	0.936
Hyperlipidemia	1.07 (0.78–1.46)	0.687	1.89 (1.28–2.79)	**0.001**
Chronic inflammatory condition	0.89 (0.51–1.55)	0.681	1.00 (0.54–1.83)	0.993
Chronic ischemic heart disease	1.24 (0.93–1.64)	0.144	1.55 (1.11–2.18)	**0.011**
Malignancy/cancer diagnosis	0.84 (0.64–1.10)	0.211	1.10 (0.81–1.49)	0.557
Depression	1.37 (1.05–1.79)	**0.019**	1.26 (0.93–1.71)	0.132
BMI (kg/m^2^)				
< 25	Ref		Ref	
25–29.9	1.39 (0.79–2.43)	0.249	1.72 (0.93–3.19)	0.0850
30–34.9	1.36 (0.78–2.36)	0.273	1.65 (0.90–3.03)	0.109
35–39.9	1.81 (1.04–3.16)	**0.037**	2.13 (1.15–3.96)	**0.016**
> 40	2.54 (1.46–4.4)	**< 0.001**	2.92 (1.58–5.42)	**< 0.001**
eGFR (mL/min)				
< 30	0.86 (0.41–1.79)	0.687	1.23 (0.56–2.69)	0.613
30–44	1.28 (0.77–2.13)	0.346	1.64 (0.93–2.90)	0.087
45–59	0.79 (0.53–1.17)	0.241	1.05 (0.68–1.64)	0.819
≥ 60	Ref		Ref	
HbA1c	1.29 (1.21–1.38)	**< 0.001**	1.32 (1.22–1.42)	**< 0.001**

*Note:* Crude odds ratios (ORs) represent unadjusted associations between each factor and medication prescription. Adjusted odds ratios (aORs) represent the association after controlling for all other variables in the model and clinic site. Clinic site coefficients are omitted for brevity but were included in all adjusted models to account for provider and regional variation. An OR > 1 indicates higher odds of receiving prescriptions for these medications, while OR < 1 indicates lower odds. Analyses based on subsample with complete BMI and eGFR data (*n* = 1678). Statistically significant associations (*p* < 0.05) are shown in bold.

## Data Availability

The analyzed data is not made publicly available due to the decision of the responsible research ethics board.

## Supporting Information

Supporting Information Additional supporting information can be found online in the Supporting Information section. Supporting information includes three tables. Table S1 compares patient characteristics between subsamples with available BMI and/or eGFR data and the overall study population. Table S2 presents separate multivariable regression analyses for key subgroups (chronic ischemic heart disease, impaired kidney function, and severe obesity). Table S3 shows factors specifically associated with SGLT2 inhibitor prescribing. The STROBE checklist for cross-sectional studies documents adherence to reporting guidelines.
