# Supplementary material for: SGLT2 Inhibitor and GLP-1 Receptor Agonist Prescriptions in Newly Diagnosed Type 2 Diabetes Patients With Cardiorenal Risks: A Cross-Sectional Study
Source: J Diabetes Res. 2025 Nov 3;2025:6656982. doi: 10.1155/jdr/6656982 (PMC12602036; doi:10.1155/jdr/6656982)
Supplement: Supporting Information — Additional supporting information can be found online in the Supporting Information section. Supporting information includes three tables. Table S1 compares patient characteristics between subsamples with available BMI and/or eGFR data and the overall study population. Table S2 presents separate multivariable regression analyses for key subgroups (chronic ischemic heart disease, impaired kidney function, and severe obesity). Table S3 shows factors specifically associated with SGLT2 inhibitor prescribing. The STROBE checklist for cross-sectional studies documents adherence to reporting guidelines. [file 6656982.f1.zip › Supplement.docx]

# Supplement

Table S1: Patient characteristics among all subsamples (p<0.05 in bold).

|  | | All^*^  *(N=5,783)* | BMI available  *(N=4,002)* | eGFR available  *(N=2,474)* | BMI & eGFR available  *(N=1,678)* | BMI  vs.  non BMI | eGFR  vs. non-eGFR | BMI & eGFR vs. non-available |
| --- | --- | --- | --- | --- | --- | --- | --- | --- |
|  |  | n (%) | n (%) | n (%) | n (%) | p^#^ | p^#^ | p^#^ |
| Sex | Male | 3,253 (56.3) | 2,272 (56.8) | 1,386 (56) | 946 (56.4) | 0.232 | 0.762 | 0.902 |
|  | Female | 2,530 (43.7) | 1,730 (43.2) | 1,088 (44) | 732 (43.6) |  |  |  |
| Age (years) | mean (SD) | 62 (14.5) | 62.9 (14.1) | 62.5 (14.7) | 63.4 (14.2) | **<0.001** | **0.023** | **<0.001** |
|  | <35 | 228 (3.9) | 136 (3.4) | 93 (3.8) | 56 (3.3) | **<0.001** | 0.093 | **<0.001** |
|  | 35 – 49 | 883 (15.3) | 544 (13.6) | 373 (15.1) | 209 (12.5) |  |  |  |
|  | 50 – 64 | 1,995 (34.5) | 1,368 (34.2) | 837 (33.8) | 589 (35.1) |  |  |  |
|  | 65 – 79 | 2,047 (35.4) | 1,501 (37.5) | 869 (35.1) | 618 (36.8) |  |  |  |
|  | 80+ | 630 (10.9) | 453 (11.3) | 302 (12.2) | 206 (12.3) |  |  |  |
| Race/ Ethnicity | Hispanic (any race) | 407 (7.1) | 261 (6.6) | 172 (7) | 111 (6.6) | **<0.001** | 0.097 | 0.630 |
|  | Non-Hispanic American Indian or Alaska Native | 27 (0.5) | 22 (0.6) | 7 (0.3) | 5 (0.3) |  |  |  |
|  | Non-Hispanic Asian | 145 (2.5) | 81 (2) | 71 (2.9) | 38 (2.3) |  |  |  |
|  | Non-Hispanic Black or African American | 357 (6.2) | 196 (4.9) | 164 (6.7) | 86 (5.1) |  |  |  |
|  | Non-Hispanic White | 4,736 (82.6) | 3,364 (84.7) | 2,004 (81.8) | 1414 (84.3) |  |  |  |
|  | Non-Hispanic Other^§^ | 64 (1.1) | 48 (1.2) | 33 (1.3) | 24 (1.4) |  |  |  |
| Type of Insurance | None | 68 (1.2) | 30 (0.7) | 24 (1) | 11 (0.7) | **<0.001** | 0.140 | **0.001** |
|  | Medicare | 2,853 (49.3) | 2,070 (51.7) | 1,260 (50.9) | 888 (52.9) |  |  |  |
|  | Medicaid | 526 (9.1) | 354 (8.8) | 218 (8.8) | 147 (8.8) |  |  |  |
|  | Other private insurance | 2,336 (40.4) | 1,548 (38.7) | 972 (39.3) | 632 (37.7) |  |  |  |
| Hypertension | | 4,547 (78.8) | 3,165 (79.3) | 1,961 (79.6) | 1344 (80.1) | 0.189 | 0.207 | 0.132 |
| Hyperlipidemia | | 4,642 (80.3) | 3,266 (81.6) | 1,961 (79.3) | 1,365 (81.3) | **<0.001** | 0.097 | 0.188 |
| Chronic inflammatory condition | | 284 (4.9) | 183 (4.6) | 135 (5.5) | 89 (5.3) | 0.074 | 0.097 | 0.377 |
| Chronic ischemic heart disease | | 1,036 (18.0) | 737 (18.5) | 509 (20.7) | 362 (21.6) | 0.134 | **<0.001** | **<0.001** |
| Malignancy/cancer diagnosis | | 1,553 (26.9) | 1,097 (27.5) | 704 (28.6) | 500 (29.8) | 0.149 | **0.014** | **0.002** |
| Depression | | 1,418 (24.6) | 984 (24.7) | 611 (24.8) | 424 (25.3) | 0.850 | 0.734 | 0.439 |

^*^ Missing values: Race/Ethnicity n=47, comorbidities n=15, ^#^ Chi-square tests used for categorical variables and Mann-Whitney *U* test for continuous variables. Statistically significant differences (p<0.05) shown in bold, ^§^ including Native Hawaiian or Other Pacific Islander

Table S2: Subgroup Analyses of Factors Associated with SGLT2 Inhibitor or GLP-1 Receptor Agonist Prescriptions. Modell further adjusted for respective primary care clinics (ORs omitted, p<0.05 in bold).

| **Factor** | | **All**  *(n=1,678)* | | **Chronic Ischemic Heart Disease**  *(n=362)* | | **Impaired Kidney Function**  *(n=343)* | | **Severe Obesity**  *(n=973)* | |
| --- | --- | --- | --- | --- | --- | --- | --- | --- | --- |
|  |  | **aOR (95% CI)** | **p** | **aOR (95% CI)** | **p** | **aOR (95% CI)** | **p** | **aOR (95% CI)** | **p** |
| Age (cont.) | | 0.99 (0.97 – 1.00) | 0.092 | 0.97 (0.93 – 1.02) | 0.215 | 0.97 (0.93 – 1.01) | 0.140 | 0.97 (0.95 – 0.99) | **0.005** |
| Sex | Male | 0.86 (0.65 – 1.14) | 0.298 | 1.36 (0.65 – 2.83) | 0.418 | 0.81 (0.37 – 1.77) | 0.596 | 0.74 (0.51 – 1.07) | 0.112 |
|  | Female | ref |  | ref |  | ref |  | ref |  |
| Race/ Ethnicity | Hispanic (any race) | 1.06 (0.63 – 1.79) | 0.833 | 4.98 (0.84 – 29.55) | 0.077 | 1.96 (0.25 – 15.14) | 0.518 | 1.04 (0.51 – 2.11) | 0.921 |
|  | Non-Hispanic American Indian or Alaska Native | 5.12 (0.79 – 33.16) | 0.087 | 0 (0 – ∞) | 1.000 | 0 (0 – ∞) | 1.000 | ∞ (0 – ∞) | 1.000 |
|  | Non-Hispanic Asian | 1.15 (0.46 – 2.85) | 0.766 | 0 (0 – ∞) | 0.999 | 0.35 (0.03 – 4.65) | 0.424 | 0 (0 – ∞) | 0.999 |
|  | Non-Hispanic Black or African American | 0.65 (0.34 – 1.26) | 0.205 | 1.02 (0.21 – 5.10) | 0.976 | 0.57 (0.12 – 2.74) | 0.479 | 0.45 (0.19 – 1.06) | 0.067 |
|  | Non-Hispanic Other | 0.27 (0.06 – 1.26) | 0.095 | 0.26 (0.02 – 4.09) | 0.340 | 0 (0 – ∞) | 0.999 | 0 (0 – ∞) | 0.999 |
|  | Non-Hispanic White | ref |  | ref |  | ref |  | ref |  |
| Type of Insurance | None | 2.29 (0.54 – 9.66) | 0.261 | ∞ (0 – ∞) | 1.000 | 0 (0 – 0) | 1.000 | 3.21 (0.42 – 24.65) | 0.262 |
|  | Medicare | 0.69 (0.47 – 1.01) | 0.053 | 0.73 (0.27 – 1.99) | 0.545 | 0.70 (0.22 – 2.20) | 0.543 | 0.9 (0.55 – 1.47) | 0.662 |
|  | Medicaid | 0.91 (0.57 – 1.47) | 0.704 | 0.79 (0.12 – 5.09) | 0.807 | 0.14 (0.01 – 1.37) | 0.091 | 1.3 (0.71 – 2.35) | 0.396 |
|  | Other private insurance | ref |  | ref |  | ref |  | ref |  |
| Hypertension | | 1.01 (0.71 – 1.46) | 0.936 | – |  | 1.85 (0.26 – 13.38) | 0.543 | 0.73 (0.46 – 1.19) | 0.207 |
| Hyperlipidemia | | 1.89 (1.28 – 2.79) | **0.001** | – |  | 3.40 (0.52 – 22.34) | 0.202 | 2.65 (1.57 – 4.47) | **<0.001** |
| Chronic inflammatory condition | | 1.00 (0.54 – 1.83) | 0.993 | 3.48 (0.43 – 28.48) | 0.244 | 0.88 (0.20 – 3.95) | 0.873 | 0.94 (0.43 – 2.08) | 0.883 |
| Chronic ischemic heart disease | | 1.55 (1.11 – 2.18) | **0.011** | – |  | 1.11 (0.51 – 2.42) | 0.796 | 1.91 (1.23 – 2.97) | **0.004** |
| Malignancy/cancer diagnosis | | 1.10 (0.81 – 1.49) | 0.557 | 0.75 (0.38 – 1.48) | 0.406 | 1.46 (0.68 – 3.11) | 0.331 | 1.12 (0.75 – 1.68) | 0.580 |
| Depression | | 1.26 (0.93 – 1.71) | 0.132 | 0.70 (0.31 – 1.57) | 0.383 | 1.14 (0.49 – 2.63) | 0.759 | 1.34 (0.91 – 1.96) | 0.133 |
| BMI (kg/m²) | <25 | ref |  | ref |  | ref |  | – |  |
|  | 25 – 29.9 | 1.72 (0.93 – 3.19) | 0.085 | 5.66 (1.03 – 31.23) | **0.047** | 3.20 (0.42 – 24.32) | 0.261 | – |  |
|  | 30 – 34.9 | 1.65 (0.90 – 3.03) | 0.109 | 3.85 (0.71 – 20.87) | 0.118 | 3.00 (0.40 – 22.61) | 0.287 | – |  |
|  | 35 – 39.9 | 2.13 (1.15 – 3.96) | **0.016** | 4.26 (0.79 – 23.07) | 0.092 | 4.26 (0.57 – 31.60) | 0.156 | – |  |
|  | >40 | 2.92 (1.58 – 5.42) | **0.001** | 4.84 (0.83 – 28.24) | 0.079 | 1.91 (0.23 – 15.64) | 0.546 | – |  |
| eGFR (mL/min) | <30 | 1.23 (0.56 – 2.69) | 0.613 | 1.17 (0.35 – 3.94) | 0.796 | – |  | 0.98 (0.30 – 3.13) | 0.967 |
|  | 30 – 44 | 1.64 (0.93 – 2.90) | 0.087 | 2.01 (0.71 – 5.69) | 0.189 | – |  | 1.23 (0.57 – 2.67) | 0.598 |
|  | 45 – 59 | 1.05 (0.68 – 1.64) | 0.819 | 0.93 (0.31 – 2.82) | 0.904 | – |  | 1.33 (0.76 – 2.34) | 0.320 |
|  | >=60 | ref |  | ref |  | – |  | ref |  |
| HbA1c | | 1.32 (1.22 – 1.42) | **<0.001** | 1.46 (1.14 – 1.87) | **0.003** | 1.89 (1.35 – 2.65) | **<0.001** | 1.28 (1.15 – 1.44) | **<0.001** |

While Table 3 presents regression results for the complete sample with BMI and eGFR data (n=1,678), this table provides separate regression analyses for three key high-risk subgroups: patients with chronic ischemic heart disease (n=362), impaired kidney function (n=343), and severe obesity (BMI>32 kg/m², n=973). All models were adjusted for the same covariates as in Table 3, with the exception of variables that define the respective subgroup (e.g., BMI categories were not included in the obesity subgroup analysis). Odds ratios represent the association between each factor and receiving prescription for these medications within the specific subgroup. Missing values for odds ratios (marked as '–') indicate that the variable was the subgroup defining characteristic.

Table S3. Factors Associated with SGLT2 Inhibitor Prescribing Among Patients with Type 2 Diabetes. Modell further adjusted for respective primary care clinics (ORs omitted, p<0.05 in bold).

| **Factor** | | **All**  *(n=1,678)* | |
| --- | --- | --- | --- |
|  |  | **aOR (95% CI)** | **p** |
| Age (cont.) | | 0.99 (0.97 – 1.01) | 0.383 |
| Sex | Male | 1.19 (0.77 – 1.84) | 0.425 |
|  | Female | ref |  |
| Race/ Ethnicity | Hispanic (any race) | 0.70 (0.29 – 1.69) |  |
|  | Non-Hispanic American Indian or Alaska Native | 1.63 (0.13 – 19.94) | 0.430 |
|  | Non-Hispanic Asian | 0.54 (0.11 – 2.57) | 0.703 |
|  | Non-Hispanic Black or African American | 0.58 (0.20 – 1.68) | 0.440 |
|  | Non-Hispanic Other | 0.34 (0.04 – 2.88) | 0.318 |
|  | Non-Hispanic White | ref |  |
| Type of Insurance | None | 0 (0 – ∞) | 0.999 |
|  | Medicare | 0.79 (0.44 – 1.42) | 0.428 |
|  | Medicaid | 0.89 (0.41 – 1.93) | 0.759 |
|  | Other private insurance | ref |  |
| Hypertension | | 1.23 (0.69 – 2.18) | 0.483 |
| Hyperlipidemia | | 2.80 (1.38 – 5.68) | **0.004** |
| Chronic inflammatory condition | | 0.66 (0.22 – 1.93) | 0.445 |
| Chronic ischemic heart disease | | 2.46 (1.52 – 3.96) | **<0.001** |
| Malignancy/cancer diagnosis | | 0.69 (0.43 – 1.13) | 0.141 |
| Depression | | 1.50 (0.95 – 2.35) | 0.082 |
| BMI (kg/m²) | <25 | ref |  |
|  | 25 – 29.9 | 1.49 (0.63 – 3.51) | 0.361 |
|  | 30 – 34.9 | 1.37 (0.60 – 3.17) | 0.457 |
|  | 35 – 39.9 | 1.06 (0.44 – 2.54) | 0.897 |
|  | >40 | 0.96 (0.39 – 2.36) | 0.923 |
| eGFR (mL/min) | <30 | 0.96 (0.31 – 2.98) | 0.938 |
|  | 30 – 44 | 1.04 (0.43 – 2.49) | 0.933 |
|  | 45 – 59 | 0.90 (0.46 – 1.78) | 0.766 |
|  | >=60 | ref |  |
| HbA1c | | 1.25 (1.12 – 1.40) | **<0.001** |
